# Efficacy and safety of a “radical” surgical strategy in the treatment of parasagittal sinus meningioma

**DOI:** 10.3389/fneur.2024.1364917

**Published:** 2024-04-08

**Authors:** Zihao Duan, Changlong Zhou, Xiaoxiao Yan, Wei Du, Xiaohui Xia, Hui Shi, Hai Su, Yi Zhang, Xuenong He, Qijiang Xiong

**Affiliations:** Department of Neurosurgery, the Affiliated Yongchuan Hospital of Chongqing Medical University, Chongqing, China

**Keywords:** sagittal sinus, meningioma, surgical strategy, microsurgery, parasagittal sinus meningioma

## Abstract

**Background:**

No standardized criteria for surgical resection of parasagittal sinus meningiomas (PSM) have been established, and different surgical strategies have been proposed. The aim of the present study was to investigate the efficacy and safety of a “radical” surgical strategy in the treatment of PSM.

**Methods:**

The clinical histories, radiological findings, pathologic features, and surgical records of 53 patients with PSM admitted by the same surgical team using the “radical” surgical strategy were retrospectively analyzed between 2018 and 2023.

**Results:**

Among the 53 PSM cases, 16 (30.2%) had a patent sinus proper, 28 (52.8%) had partial obstruction of the sinus proper, and 9 (17.0%) had complete obstruction of the sinus proper before the operation. During operation, Simpson grade I resection was performed in 34 (64.2%) cases and Simpson grade II in 19 (35.8%) cases. Postoperative pathologic examination suggested tumors of WHO grade I in 47 (88.7%) cases, WHO grade II in 4 (7.5%) cases, and WHO grade III in 2 (3.8%) cases. Postoperative complications primarily included a small amount of delayed intracerebral hemorrhage in 3 (5.7%) cases, exacerbation of cerebral edema in 3 (5.7%) cases, exacerbation of motor and sensory deficits in 4 (7.5%) cases, and intracranial infection in 2 (3.8%) cases. There were no cases of death or new-onset neurological dysfunction. Dizziness and headache symptoms improved to varying degrees, and a seizure-free status was achieved postoperatively. Excluding one case lost to follow-up, the average follow-up period was 33 months, and there were no cases of recurrence.

**Conclusion:**

A “radical” strategy for the surgical management of PSM is effective, safe, and simple to perform, provided that the sagittal sinus is properly managed and its associated veins are protected.

## 1 Introduction

Parasagittal sinus meningioma (PSM) was first described by Cushing and Eisenhardt (1) as a tumor that occupies the parasagittal angle of the sagittal sinus, with no brain tissue between the tumor and the sagittal sinus. PSMs may also partially or completely invade the sagittal sinus. PSM accounts for 20%–30% (2) of all meningiomas and 25% of the meningiomas seen in our department during the same period. Although meningiomas grow slowly and are generally well-defined, PSMs tend to invade the superior sagittal sinus to varying degrees and are adjacent to many cortical veins and major functional areas. This greatly increases the difficulty of complete surgical resection, making radical resection of PSMs without complications and recurrence a major challenge for neurosurgeons.

Given these difficulties and risks, no consensus on the surgical management of PSM currently exists. A literature review showed that surgical strategies can be broadly categorized as “radical” (3–8) or “cautious” (2, 9–11). “Radical” resection strategies typically consist of resecting the tumor inside and outside the sagittal sinus, followed by ligation and division, suture repair, or reconstruction of the sagittal sinus, to enhance the likelihood of achieving surgical cure. “Cautious” resection strategies typically focus on intraoperative protection of the venous sinuses and only involve resection of the tumor outside the sinus without operating on the part inside the sinus, followed by observation, radiotherapy, or re-operation; however, there is an increased risk of tumor residue and recurrence.

In this study, we adopted a “radical” surgical strategy for 53 patients with PSM at our center and achieved satisfactory results. We summarize and detail the surgical techniques and perioperative management of PSM with different degrees of sinus invasion.

## 2 Materials and methods

A total of 53 cases of PSM treated by the same surgical team using a “radical” surgery strategy at the Department of Neurosurgery of Yongchuan Hospital, Chongqing Medical University were collected between 2018 and 2023. MRI (plain + enhanced + MRV) and CT were performed at the time of admission; some patients also underwent CTV or DSA. The lesions were on the right side of the sinus in 31 (58.5%) cases and on the left side in 22 (41.5%) cases. The maximum size was 7.7 × 6.5 × 6.7 cm, the minimum size was 1.6 × 1.4 × 1.0 cm, and the mean size was 4.0 × 3.6 × 4.0 cm. There were 23 (43.4%) cases accompanied with peritumoral brain edema, of which 7 (13.2%) cases were severe. PSM was broadly classified into three types based on the degree of sinus invasion. Type I (patent) tumors adhere to or invade only the outer layer of the sagittal sinus wall and do not affect the sinus proper; this is comparable to Sindou type I. Type II (partial obstruction) tumors have broken through the outer layer of the sagittal sinus wall and are pushing or growing into the sinus proper, resulting in partial obstruction of the sinus cavity; this includes Sindou types II-IV. Type III (complete obstruction) tumors involve two or all walls of the sagittal sinus with complete obstruction of the sinus proper and includes Sindou types V and VI. General characteristics and preoperative clinical symptoms concerning 53 patients with PSM are summarized in Tables 1, 2, respectively.

**Table 1:** General characteristics of the 53 patients with PSM.

Parameter	Frequency (%)
Number of patients	53
Age (years, mean ± SD)	53.4 ± 14.1
Primary: Recurrent	53:0
Sex (male: female)	22:31(1:1.4)
Mean tumor size	4.0 × 3.6 × 4.0 cm
**Tumor site**	
Anterior 1/3 of SSS^*^	12(22.6%)
Middle 1/3 of SSS	25(47.2%)
Posterior 1/3 of SSS	11(20.8%)
Confluence of sinuses	5(9.4%)
**Extent of SSS invasion**	
Type I^*^ (patent)	16(30.2%)
Type II^*^ (partial obstruction)	28(52.8%)
Type III^*^ (complete obstruction)	9(17.0%)
**WHO pathological grade**	
I	47(88.7%)
II	4(7.5%)
III	2(3.8%)

^*^SSS, superior sagittal sinus; type I equivalent to Sindou type I, type II equivalent to Sindou types II–IV, type III equivalent to Sindou types V–VI.

**Table 2:** Preoperative clinical symptoms.

Site	Anterior 1/3 of SSS	Middle 1/3 of SSS	Posterior 1/3 of SSS	Confluence of sinuses	Total
Symptom					
Dizziness, headache	9(75.0%)	14(56.0%)	7(63.6%)	5(100.0%)	35(66.0%)
Cognitive dysfunction	4(33.3%)	6(25.0%)			10(18.9%)
Numbness (lower and/or upper limbs)		7(28.0%)			7(13.2%)
Fatigue (lower and/or upper limbs)		8(32.0%)			8(15.1%)
Generalized seizures	3(25.0%)	2(8.0%)			5(9.4%)
Focal seizures		4(16.0%)	1(9.1%)		5(9.4%)
Decreased vision	3(25.0%)	2(8.0%)	2(18.2%)	3(60.0%)	10(18.9%)
Asymptomatic	1(8.3%)	5	2		8

The study was approved by the Medical Research Ethics Committee of Yongchuan Hospital, Chongqing Medical University (no. 2023-KLS-65).

### 2.1 “Radical” surgical strategy

#### 2.1.1 Management of sinuses and associated veins with tumor involvement

**Figure 1:**
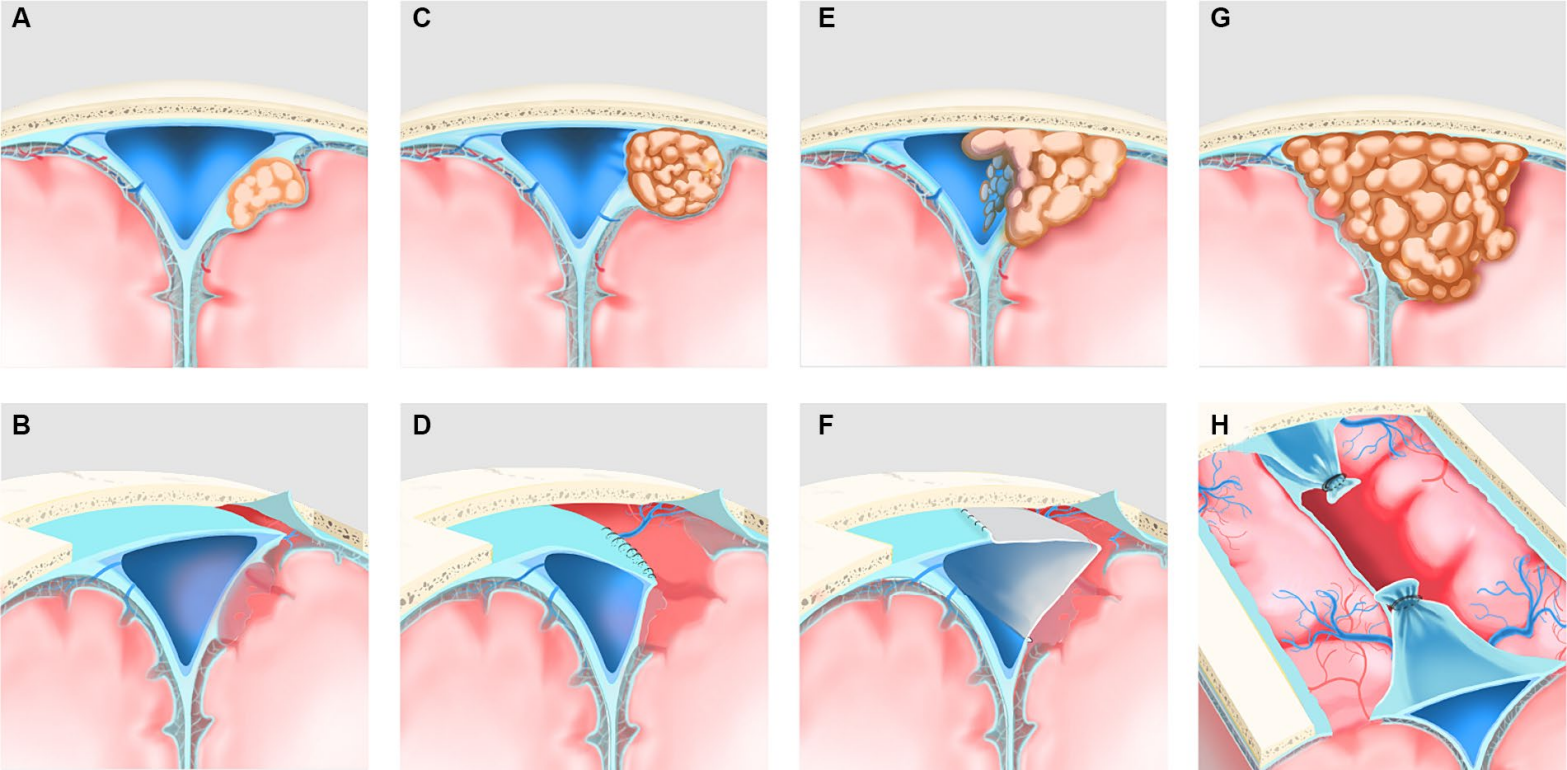
Reclassification of PSM and its corresponding surgical methodologies was illustrated. **(A)** Type I (patent) preoperatively, the tumor invaded the outer layer of the ipsilateral sagittal sinus wall only and did not affect the sinus proper. **(B)** Postoperatively, the extra-sinus tumor and the outer layer of the sinus wall, which was involved owing to the tumor, were resected. **(C)** Type II (partial obstruction) preoperatively; the tumor grew into the lateral recess and only pushed the sinus cavity, resulting in mild obstruction of the sinus proper. **(D)** Postoperatively, the lateral recess was cut open, the extra- and intra-sinus tumor was resected, and the roof of the sinus and the ipsilateral wall were then closed with sutures. **(E)** Type II (partial obstruction) preoperatively, the tumor grew into the sinus cavity and invade the one or both of the two sinus walls with more severe obstruction of the sinus proper. **(F)** Postoperatively, the sinus wall involved owing to the tumor, and the extra- and intra-sinus tumor were resected, and sinus repair or sinus reconstruction was performed using a patch. **(G)** Type III (complete obstruction)preoperatively, the tumor involved two or all walls of the sagittal sinus with complete obstruction of the sinus proper. **(H)** Postoperatively, the sinus involved owing to the tumor was ligated proximally and distally, and the sinus and the extra- and intra-sinus tumor were resected.

For type I, the outer layer of the sinus wall was resected while keeping the sinus proper intact, and a gelatin sponge or muscle paste was used to cover the defect and immobilize it with “bio-protein gel” (Figures 1A,B). For type II, if the tumor grew into the lateral recess and only compressed the sinus cavity, the lateral recesses were cut open, the tumor was resected, and the roof of the sinus and the lateral wall were then closed with sutures (Figures 1C,D). If the tumor had grown into the sinus proper and invaded the one or both two sinus walls, the sinus wall and the tumor inside the sinus were resected, and sinus repair or sinus reconstruction was performed using autologous dura mater, the temporal fascia, or artificial meninges (Figures 1E,F). For type III, the sinus was ligated proximally and distally, and the sinus and the tumor inside the sinus were resected (Figures 1G,H). Patch repair for reconstruction of the sinus cavity could also be considered if poor venous compensation is observed.

#### 2.1.2 Management of tumors involving the skull

If the tumor only caused local hyperplasia or destruction of the inner table of the skull, the skull was ground with a bur to remove the diseased bone and completely cauterized using an electric knife. If the tumor had caused serious changes in the skull, then that part of the skull was removed, and a titanium mesh or other material was used to repair the cranial defect.

#### 2.1.3 Management of tumors near the arachnoid, pia mater, or brain tissue

Tissues were resected together in cases of tumor involvement.

#### 2.1.4 Management of the tumor base

The dura of the tumor base, which includes 2 cm of normal dura outside the tumor, was resected.

### 2.2 Postoperative observation and evaluation

Postoperatively, the patient was admitted to the neurosurgical intensive care unit for management for 1–3 days, and cranial CT was performed immediately after resuscitation and respirator weaning. The first MRI (plain + enhanced + MRV) examination was completed within 3 days postoperative. Surgical outcomes were assessed clinically and by imaging.

### 2.3 Follow-up

Radiological follow-up and clinical evaluation were performed at 3, 6, and 12 months postoperative and annually thereafter. The follow-up interval was shortened for patients who underwent sinus wall resection with patch reconstruction.

## 3 Results

**Figure 2:**
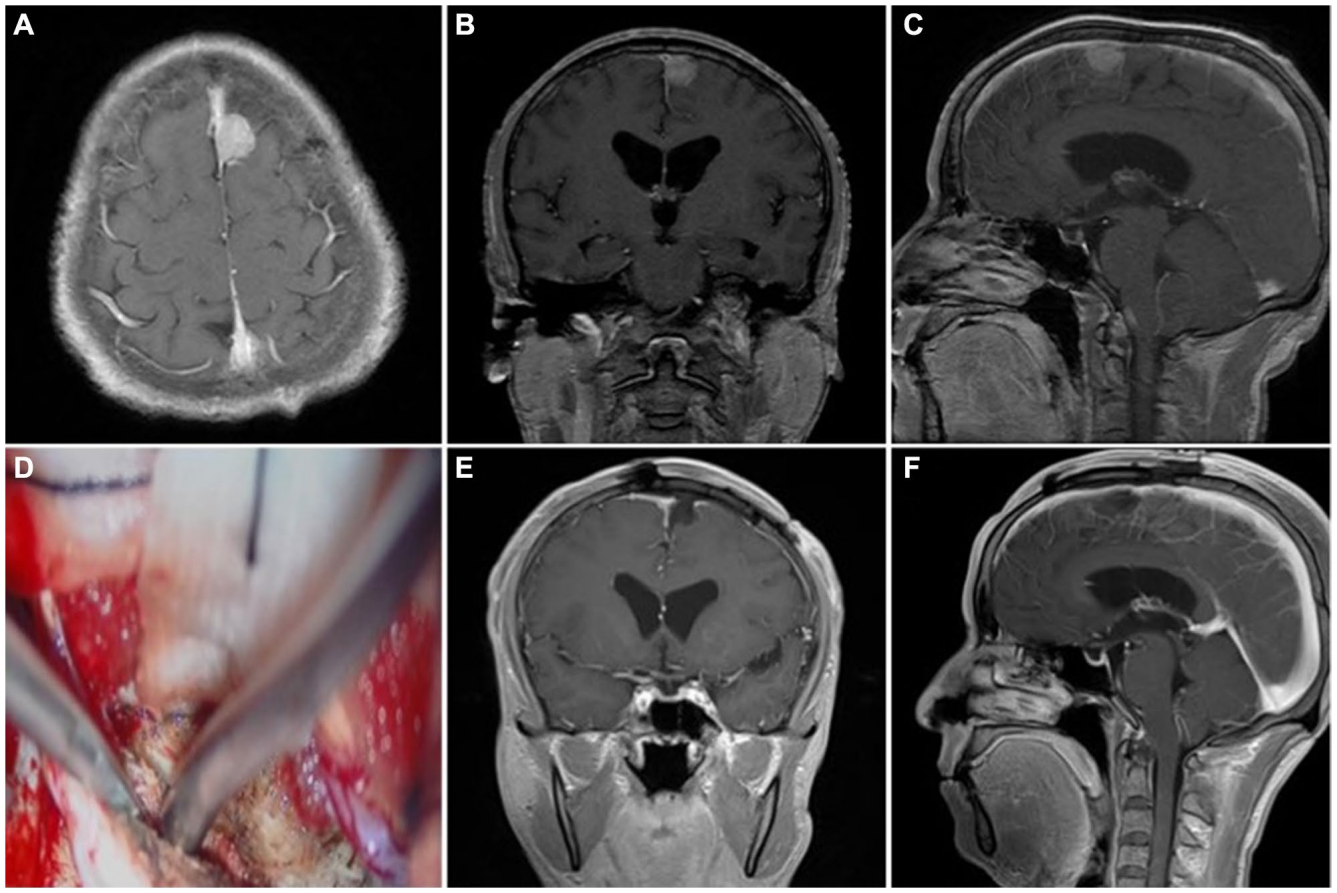
Representative case 1, type I (patent sinus). **(A–C)** Preoperative enhanced MRI images. **(D)** Intraoperative resection of the outer layer of the sinus wall. **(E,F)** Postoperative enhanced MRI images.

**Figure 3:**
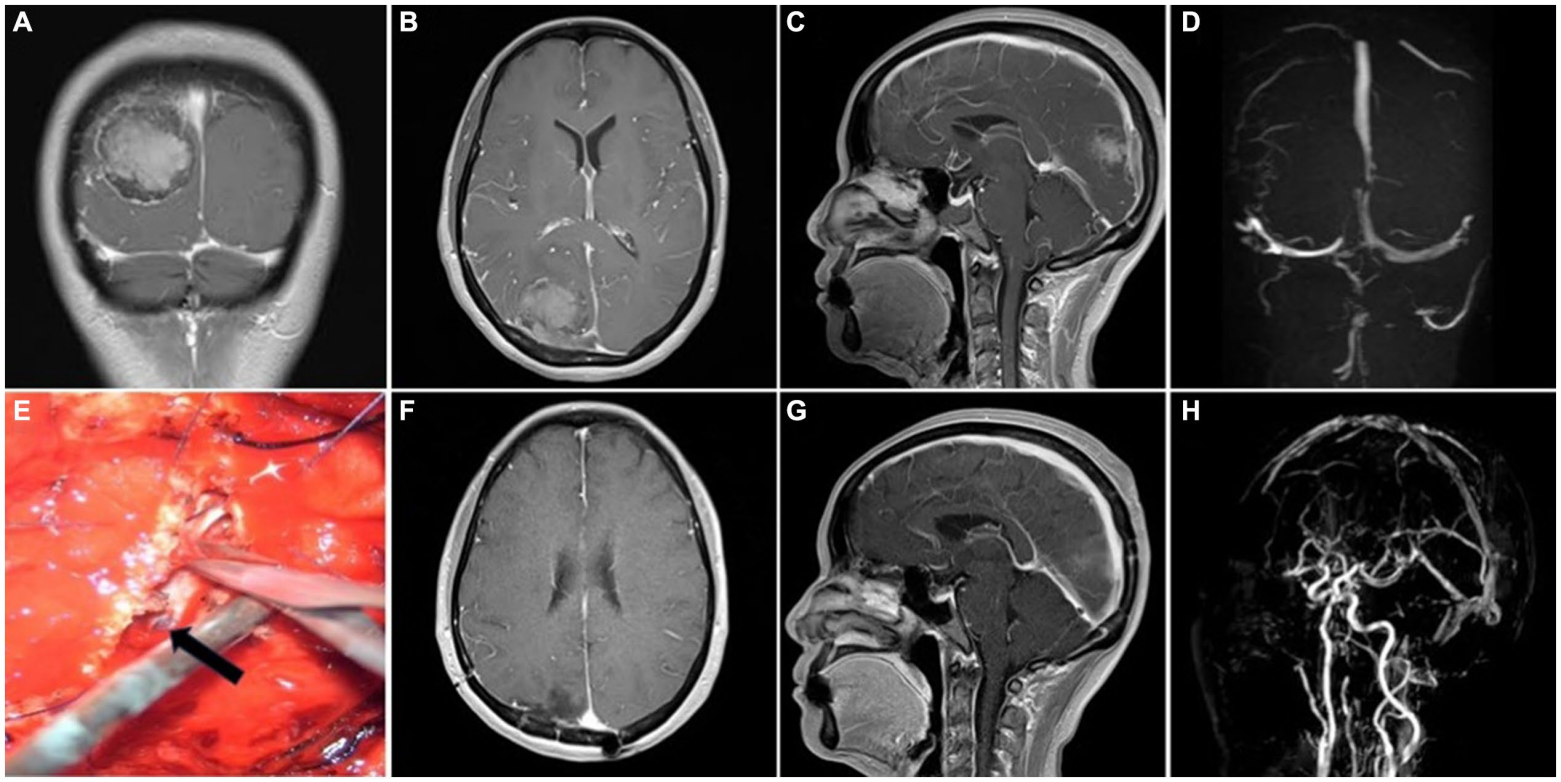
Representative case 2, type II (partial sinus obstruction). **(A–C)** Preoperative enhanced MRI images. **(D)** Preoperative MRV images. **(E)** Intraoperative suture repair of the sinus wall. The arrow shows the intact inner layer compressed by tumor. **(F,G)** Postoperative enhanced MRI images. **(H)** Postoperative MRV images.

**Figure 4:**
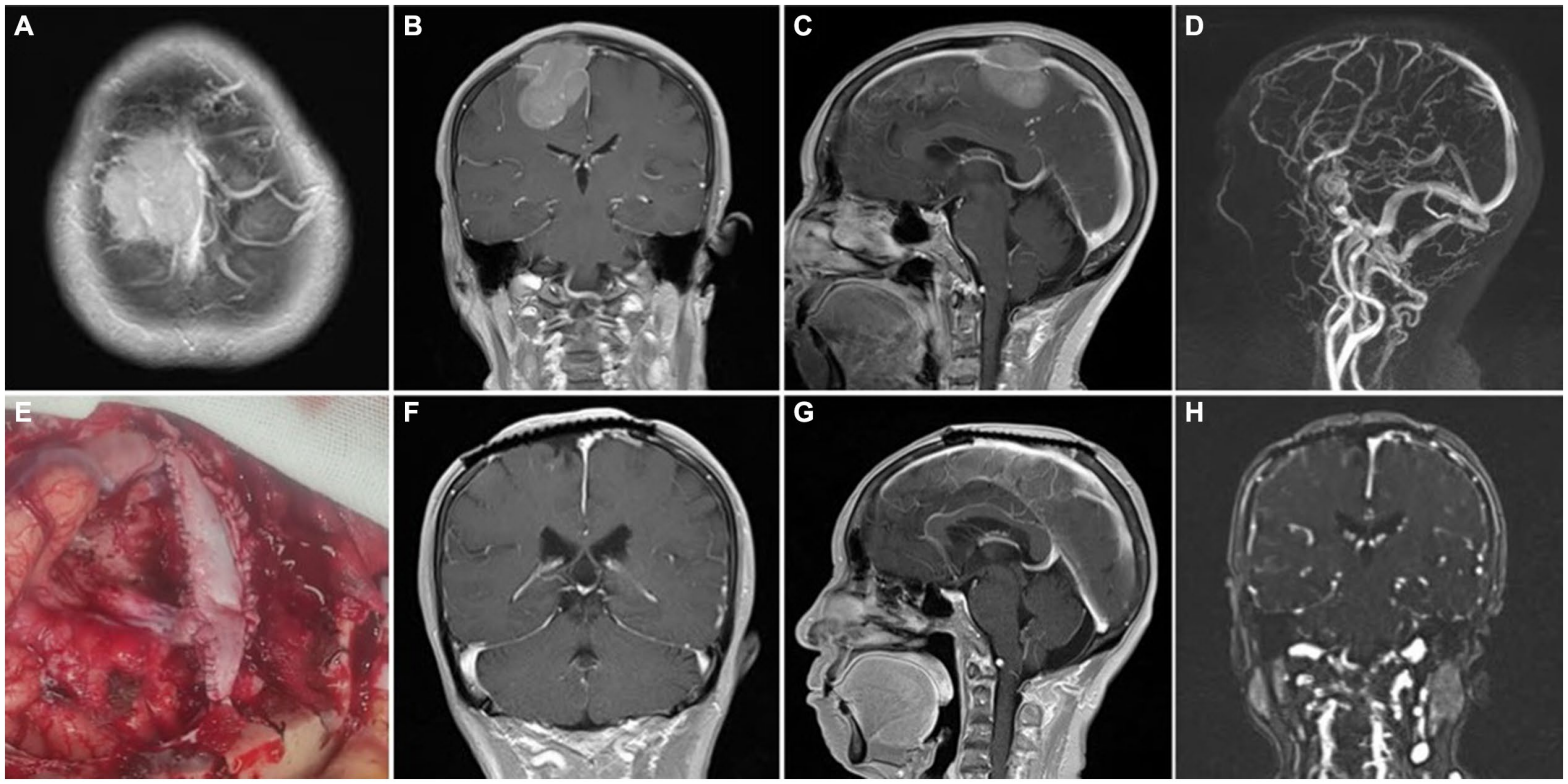
Representative case 3, type III (complete sinus obstruction). **(A–C)** Preoperative enhanced MRI images. **(D)** Preoperative MRV images. **(E)** Intraoperative patch reconstruction of the sinus wall. **(F,G)** Postoperative enhanced MRI images with concurrent titanium mesh repair of the cranial defect. **(H)** Postoperative MRV images.

**Figure 5:**
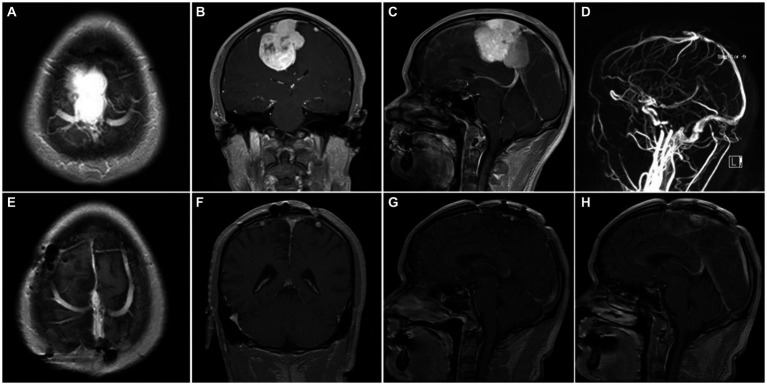
Representative case 4, type III (complete sinus obstruction). **(A–C)** preoperative enhanced MRI images. **(D)** preoperative MRV images. **(E–H)** Postoperative enhanced MRI images. Intraoperative sinus ligation and division without sinus reconstruction.

Based on the Simpson grade of meningioma resection (12), all 53 cases in the cohort underwent complete resection under microscopy and MRI confirmation within 3 days postoperative. Simpson grade I resection was achieved in 34 (64.2%) cases, and Simpson grade II resection was achieved in 19 (35.8%) cases. Among the patients presenting with preoperative symptoms of cognitive dysfunction, limb numbness or fatigue, and vision loss, 33 (62.3%) patients demonstrated postoperative improvement, while 20 (37.7%) patients continued to experience persistent symptoms (Table 3). Postoperative dizziness and headache symptoms improved to varying degrees, and a seizure-free status was achieved. The outer layer of the sinus wall was resected in 14 (26.4%) patients (a representative case is illustrated in Figure 2) and electrocoagulation only occurred in 2 (3.8%) patients. The sinus wall was directly sutured in 13 (24.5%) patients, all exhibiting mild stenosis (Figure 3). Patch reconstruction was performed in 20 (37.7%) patients, with sinus patency confirmed using MRV in all cases (Figure 4). Sinus ligation and division were performed in 4 (7.6%) patients (Figure 5).

**Table 3:** Surgical outcomes.

Extent of SSS invasion	Cases	Grade of resection		Neurological function	
		Simpson I	Simpson II^*^	Improvement	Same as preoperative
Type I (patent)	16	14	2	11	5
Type II (partial obstruction)	28	16	12	19	9
Type III (complete obstruction)	9	4	5	3	6

^*^Simpson grade II resection is defined as electrocoagulation only with complete or partial preservation of the sinus wall to avoid affecting the collateral veins.

There were no cases of perioperative death. There were 3 (5.7%) cases of small late-onset intracerebral hemorrhage after heparin anticoagulant therapy, which did not increase after heparin was discontinued; 3 (5.7%) cases of postoperative exacerbation of cerebral edema and symptoms of cranial hypertension, which gradually improved with dehydration, protein, and hormones; 4 (7.5%) cases of postoperative exacerbation of limb numbness and paralysis, which recovered in 1 month in conjunction with rehabilitation therapy; and 2 (3.8%) cases of intracranial infections, which recovered after antibiotic treatment. There were no definitive cases of air embolism and MRV-confirmed venous sinus thrombosis. Pathologic findings suggested tumors of WHO grade I in 47 (88.7%) cases, WHO grade II in 4 (7.5%) cases, and WHO grade III in 2 (3.8%) cases.

Excluding one patient lost to follow-up, 52 patients were followed up through June 2023 for a total ranging from 6 to 63 months (mean follow-up 33 months). There were no cases of recurrence.

## 4 Discussion

In a seminal paper published in 1957, Simpson et al. (12) introduced a classification system consisting of five levels for meningioma resection and provided detailed definitions for each level, elucidating the relationship between the extent of tumor resection and its recurrence. This correlation has since formed the theoretical foundation underlying the aggressive surgical treatment strategy for meningiomas. However, parasagittal sinus meningiomas are often located in critical intracranial areas, and surgical procedures are prone to damage important cortical areas, the superior sagittal sinus, and its important collateral draining veins, which are often accompanied by various degrees of complications, such as limb paralysis, severe cerebral edema, and even death.

To mitigate these risks, researchers have adopted a surgical strategy of “conservative resection,” but a large body of evidence suggests that meningioma recurrence is strongly correlated with the degree of resection. Conservative resection of PSM is associated with an increased recurrence rate (3, 5, 6), emphasizing the importance of radical resection. Management of the tumor invading the sinus and the associated veins is essential for achieving radical resection of PSM. Sindou et al. (3) found that repair and reconstruction of the sinus after radical tumor resection or restoration of sinus circulation by a bypass resulted in a recurrence rate of only 4%. Zhang et al. (8) reported a recurrence rate of 0% and a postoperative complication rate of 7.2% after radical tumor resection and sinus reconstruction with a simplified surgical strategy. All complications were treated aggressively, and all patients recovered uneventfully.

Our team has formulated a “radical surgery strategy” for PSMs based on “Simpson Grade I Resection Criteria” for meningiomas, especially the corresponding sinus management strategy according to the degree of sinus invasion of PSMs, which is easy to implement during surgery, does not significantly increase surgical risk and postoperative complications, and has shown remarkable therapeutic results. To improve the surgical management of PSMs, we believe that the following issues should be considered.

### 4.1 Assessment, identification, and protection of the sagittal sinus, tributary veins, and collateral veins

Protection of the venous circulation is known to be critical to surgical success for PSMs. Preoperative prognosis combined with intraoperative reality or intraoperative evaluation, utilizing techniques such as indocyanine (ICG) (13) video angiography, is required. First, a detailed preoperative evaluation of the cerebral veins based on DSA, CTV, or MRV imaging was performed. In particular, MRV is increasingly becoming the preferred option for visualization of intracranial veins because it is non-invasive and radiation-free. However, preoperative imaging of the sinus does not always match the actual condition of the sinus, so intraoperative biopsy or opening of the sinus wall is recommended for exploration to determine whether the sinus is completely obstructed. Furthermore, it is not recommended to perform direct ligation and resection of the superior sagittal sinus without assessing whether the local drainage vein is well compensated, even if preoperative imaging indicates type III. When preoperative symptoms of venous hypertension are under consideration, along with imaging indicating a scarcity of side drainage veins around the tumor, characterized by small venous vessel diameters and a singular drainage pattern, or when peritumoral brain edema (PTBE) coincides with poor brain-tumor interface-related edema (PIRE) (14, 15), there is a likelihood of insufficient local compensatory drainage veins. Hence, reconstruction of the sinus cavity warrants consideration (Figure 4). Conversely, we believe excision of the sinus without reconstruction is safe (Figure 5). Increased tumor size is accompanied by compression of the cerebral cortex and cortical bridging veins, which usually exhibit pathological changes upon compression by the tumor, resulting in poor venous return. The collateral venous circulation will gradually develop and play an important role in venous return. Destruction of veins during the procedure may lead to several adverse consequences, such as cerebral infarction or hemorrhage, seizures or neurological deficits, and even death in severe cases. Therefore, protecting the peritumoral veins is critical to the success of the procedure (16, 17). Intraoperatively, it is necessary to open the dura mater from above the tumor to avoid damaging the cortical veins at the tumor margins and to fully decompress the tumor. Then, the margins of the tumor are isolated after a decrease in peritumoral venous tension and relaxation of the tumor-brain interface. Finally, tumor veins must be accurately identified, and all preservable normal veins must be protected.

### 4.2 Control of bleeding after opening the sinus wall, as well as prevention and monitoring of air embolism

In our experience, the sinus wall is opened from the proximal to the distal end, and a single gelatin sponge was applied immediately afterward to stop proximal hemorrhage; gelatin sponge fragments should not be used to prevent leaving residue in the sinus. In addition, the tumor is resected from the proximal to the distal end, and the sinus wall is sutured or repaired while gradually advancing until the tumor is resected and the sinus is reconstructed. The risk of air embolism varies among case reports and is affected by surgical position. Air embolism was diagnosed in only one of the 100 patients (1%) reported by Alvernia and Sindou (18), although most patients were in a semi-sitting position. The management of air embolism is primarily focused on prevention, with particular emphasis placed on ensuring that the operative area is positioned below the level of the heart when incising the sinus wall. After the proximal sinus wall was opened, the surgical assistant immediately closed the defect with a gelatin sponge and resected the tumor toward its distal end while ensuring that the proximal end remained closed. When the reconstruction was completed, the last of the residual tumor in the distal sinus was resected, and blood flowed from the distal end, confirming adequate perfusion of the reconstructed sinus segment and overflowing from the distal end (terminal pinhole). Then, the sinus was closed by tightening the suture. Currently, no effective monitoring procedures are available for early identification of air embolism. Indicators of air embolism can appear on transesophageal ultrasound or various anesthesia monitoring systems but with a clear time lag.

### 4.3 Prevention and treatment of venous sinus thrombosis

Although most meningiomas are benign tumors, they are strongly associated with systemic thromboembolic states. The intrinsic biological activity of meningiomas exerts local and systemic hormonal and hematologic effects that play a major role in venous sinus thrombosis. In particular, parasagittal sinus meningiomas are more prone to promote local thrombosis because of their complex connections with vascular structures (4). In addition, excessive intraoperative manipulation of the venous sinus and sacrifice of peritumoral veins can promote thrombosis in the injured vessels. Currently, the preferred imaging modality for diagnosing venous sinus thrombosis is MRV, which is highly sensitive, non-invasive, and free of radiation, making it favored by many physicians and patients (19). The need for anticoagulants in the management of venous sinus thrombosis has been underscored by many researchers in the literature. Sindou et al. (3) concluded that anticoagulant therapy is required for at least 3 months after radical resection, a strategy that does not increase the rate of bleeding complications. A review of 34 studies on the interventional management of venous sinus thrombosis by Medel et al. (20) concluded that systemic anticoagulant therapy is a rational initial strategy for the treatment of venous sinus thrombosis, even in the presence of cerebral hemorrhage. A retrospective study of patients with cerebellopontine angle tumors by Moore et al. (21) found that five of the 43 patients developed thrombosis of the transverse and sigmoid sinuses after tumor resection. All patients with thrombosis were treated with intravenous heparin without any complications, allowing us to conclude that initiating anticoagulant therapy early in the postoperative period is safe and effective and prevents the progression of venous sinus thrombosis. Similar to other researchers, we hypothesized that patients undergoing patch repair or venous sinus reconstruction may have a higher incidence of venous sinus thrombosis due to the surgical procedure simultaneously compromising the original support of dural structures at the three corners of the sagittal sinus, rendering the sinus cavities more vulnerable to factors such as blood pressure, blood volume, blood viscosity, intracranial pressure, and cerebral edema. Therefore, meticulous management of these aforementioned factors is imperative during the postoperative period. We routinely implemented low molecular weight heparin anticoagulant therapy after the initial CT re-examination to rule out intracranial hemorrhage, during which the patient was closely observed for neurological symptoms and gastrointestinal, oral, and skin mucosal bleeding. In addition, coagulation function, thromboelastography, coagulation index, and other indicators were monitored with detailed follow-up. Following discharge, appropriate oral antiplatelet or anticoagulant medications were administered for 3–6 months post-operation. In the present cohort, anticoagulant therapy was applied in 20 cases of patch reconstruction and 13 cases of sinus wall suturing. There were no cases of hemorrhage other than only 3 cases of small delayed intracerebral hemorrhage in the edematous area outside the tumor, and there were no cases of venous sinus thrombosis.

### 4.4 Innovations of this study

This study has several innovations compared with other studies. The “radical” surgical strategy for PSMs is described in detail, and all the technical details of this surgical strategy, including the management of tumors with different types of sinus involvement, the method of sinus reconstruction, and the control of intraoperative hemorrhage are described in detail so that our treatment model is clear and easy to understand. Additionally, the details of the venous reconstruction technique are relatively simple and do not require complicated operations, such as the use of autologous saphenous vein bypass, making the approach to venous sinus reconstruction easier to implement.

### 4.5 Limitations

The follow-up period of this study was limited, so the long-term effectiveness of our “radical” surgical strategy must be confirmed with a longer follow-up period. Given that our surgical approach was as aggressive as that reported by Sindou et al. (3), we predict that the long-term recurrence rate of our procedure will be comparable to theirs, at approximately 4%. At the same time, we recognize that our sample size is relatively small compared to other studies, which may affect our judgment of the long-term effects of surgical strategies. Therefore, the sample size should be increased in future studies to facilitate a more in-depth, comprehensive, and accurate investigation of the long-term efficacy and safety of this surgical strategy.

## 5 Conclusion

A “radical” surgical strategy for increasing the degree of parasagittal sinus meningioma resection and reducing the rates of residual tumor and recurrence is effective, safe, and simple to perform, provided that the sagittal sinus is properly managed and its associated veins are protected.

## Data availability statement

The original contributions presented in the study are included in the article/supplementary material, further inquiries can be directed to the corresponding author.

## Ethics statement

The studies involving humans were approved by Medical Research Ethics Committee of Yongchuan Hospital, Chongqing Medical University. The studies were conducted in accordance with the local legislation and institutional requirements. The participants provided their written informed consent to participate in this study. Written informed consent was obtained from the individual(s) for the publication of any potentially identifiable images or data included in this article.

## Author contributions

ZD: Data curation, Formal analysis, Investigation, Methodology, Software, Validation, Writing – original draft, Writing – review & editing. CZ: Conceptualization, Funding acquisition, Resources, Supervision, Writing – review & editing. XY: Writing – review & editing. WD: Investigation, Writing – review & editing. XX: Methodology, Writing – review & editing, Formal analysis. HSh: Methodology, Writing – review & editing. HSu: Conceptualization, Writing – review & editing. YZ: Conceptualization, Writing – review & editing. XH: Conceptualization, Writing – review & editing. QX: Conceptualization, Methodology, Writing – review & editing, Formal analysis, Investigation, Project administration, Supervision, Validation, Writing – original draft.
